# A novel ultrasound image diagnostic method for thyroid nodules

**DOI:** 10.1038/s41598-023-28932-2

**Published:** 2023-01-30

**Authors:** Zhiqiang Zheng, Tianyi Su, Yuhe Wang, Zhi Weng, Jun Chai, Wenjin Bu, Jinjin Xu, Jiarui Chen

**Affiliations:** 1 College of Electronic and Information Engineering, Inner Mongolia University, Hohhot, 010021 China; 2 Department of Imaging Medicine, Inner Mongolia People’s Hospital, Hohhot, 010017 China; 3 Department of Ultrasound Medicine, Inner Mongolia People’s Hospital, Hohhot, 010017 China

**Keywords:** Computational biology and bioinformatics, Oncology, Signs and symptoms

## Abstract

The incidence of thyroid nodules is increasing year by year. Accurate determination of benign and malignant nodules is an important basis for formulating treatment plans. Ultrasonography is the most widely used methodology in the diagnosis of benign and malignant nodules, but diagnosis by doctors is highly subjective, and the rates of missed diagnosis and misdiagnosis are high. To improve the accuracy of clinical diagnosis, this paper proposes a new diagnostic model based on deep learning. The diagnostic model adopts the diagnostic strategy of localization-classification. First, the distribution laws of the nodule size and nodule aspect ratio are obtained through data statistics, a multiscale localization network structure is a priori designed, and the nodule aspect ratio is obtained from the positioning results. Then, uncropped ultrasound images and nodule area image are correspondingly input into a two-way classification network, and an improved attention mechanism is used to enhance the feature extraction performance. Finally, the deep features, the shallow features, and the nodule aspect ratio are fused, and a fully connected layer is used to complete the classification of benign and malignant nodules. The experimental dataset consists of 4021 ultrasound images, where each image has been labeled under the guidance of doctors, and the ratio of the training set, validation set, and test set sizes is close to 3:1:1. The experimental results show that the accuracy of the multiscale localization network reaches 93.74%, and that the accuracy, specificity, and sensitivity of the classification network reach 86.34%, 81.29%, and 90.48%, respectively. Compared with the champion model of the TNSCUI 2020 classification competition, the accuracy rate is 1.52 points higher. Therefore, the network model proposed in this paper can effectively diagnose benign and malignant thyroid nodules.

## Introduction

The incidence of thyroid nodules in the population rises year by year, and ultrasound is an important methodology that is currently used in the diagnosis of thyroid cancer because it is noninvasive, safe, and economical. However, ultrasonic images have disadvantages, such as low contrast, low resolution and ease of being polluted by noise, and the rates of missed diagnosis and misdiagnosis by doctors are higher^1^. Therefore, some scholars use traditional machine learning algorithms^2–4^ to analyze and process ultrasonic images, but this requires the artificial design of feature extraction algorithms, so they are weak and difficult to deploy on large-scale medical data. In contrast, deep learning carries out big data training through the construction of a deep convolutional neural network, and the network learns autonomously and is robust.

Research on deep learning in the ultrasonic diagnosis of thyroid nodules has focused mainly on improving feature extraction ability and feature diversification. The enhancement of feature extraction ability is mainly achieved by improving the network structure. Based on the structure of YOLOv2^5^, reference^6^ integrates the extracted low-level features and high-level semantic features through channel splicing to obtain better fine-grained features. However, this YOLO series network is focused on realizing fast detection, and it has difficulty meeting the accuracy requirements for practical applications. In reference^7^, a nodular region image is obtained by locating the nodular region and cropping, and a nodule edge image is extracted from the cropped image and combined for classification. However, the number of layers in the classification network is too small to effectively extract the nodule features. The scheme in^8^ used two different feature extractors to build a dual-branch classification network, which enhances the feature extraction ability of the network, but the network does not extract the features of the nodule area in a targeted manner. The approach devised in^9^ fuses deep semantic features with shallow texture features, which has a higher classification accuracy than using a single feature, but the traditional machine learning algorithm used to extract texture features is difficult to adapt to complex and changeable ultrasound data. In reference^10^, two VGG networks are used to build a cascade network, and a secondary classification is carried out for the difficult-to-classify nodules. However, the used data set is class-imbalanced, making the network less sensitive. Literature^11^ proposes the use of a fully convolutional segmentation network to obtain the region of interest and generate a classification training dataset. Then, the prediction results are output by an integrated model obtained by fusing three DenseNets, and the classification performance of the model is improved. The approach conceived in^12^ uses dual-branch networks to obtain the original image features of nodules and the texture features of the regions of interest of the nodules and then spliced the two features to classify the nodules. Reference^13^ combined transfer learning with online learning, showing that ultrasound-based transfer learning can improve the accuracy of model recognition, and designs a multi-ROI region feature fusion model. Although their model can effectively improve the classification accuracy, it requires manual extraction of regions of interest for nodules. However, the region of interest dataset still needs to be obtained manually in the data preprocessing stage. Feature diversification is realized by extracting data differences. The approach developed under^14^ extracts features from conventional ultrasound imaging and ultrasound elastography of nodules to obtain a mixed pathological feature space and complete the classification. However, only high-level features are used in the experiment, and low-level features of different images are not fully considered. Reference^15^ uses the difference in imaging perspective to extract features from images from different angles of the same nodule, and uses an attention network to achieve feature diversification, thereby improving the accuracy of network classification. However, the attention network only acts on high-level semantic features and does not give attention to low-level textures, so clinical features of nodules are easily lost. In literature^16^ uses dynamic contrast-enhanced ultrasound (CEUS) imaging to diagnose nodules and combines dynamic enhanced feature learning with hierarchical nodule classification to effectively improve the accuracy of nodule identification, but its dataset is too small, and the network is too large. However, there is a risk of overfitting. The scheme in^17^ combines the original images of nodules with local binary patterns (LBP) images and discrete wavelet transform (DWT) images and introduced expert experience to classify nodules, but the dataset is classified as benign and malignant by TI-RADS grading, not by fine needle aspiration (FNA). Reference^18^, transfer learning was applied to radiomics, which improved the generalization ability of the model and reduced the amount of training parameters. At the same time, the hyperparameter optimization based on the simulated annealing algorithm largely ensures that the transfer learning radiomics converge to an optimal hyperparameter combination. In the literature^19^, a series of clinical factors were obtained and digitized by physician assessment and fused with features extracted by an ultrasound image feature extraction network. They are fed into a fully connected network with convolutional features and finally the prediction results are obtained.

However, due to the poor quality of medical ultrasound imaging and the lack of obvious image differences between the nodules and the surrounding tissue^20^, it is not conducive to the network extracting more effective features, and the classification performance is impaired. Therefore, it is necessary to improve the feature extraction ability of the network. The channel attention mechanism^21^ is applied as an effective measure, which is characterized by mapping scalar weights on each channel dimension from feature graphs and multiplying the feature graphs and weights to carry out adaptive weighting of the features. However, each weight is obtained by the global pooling of features, and the corresponding feature is not fully utilized. Therefore, based on channel attention, asymmetric sparse convolution is used to enhance the attention mechanism in the spatial dimension to reduce the information loss caused by global pooling, to obtain global sparse attention.

In addition to improving the network structure and enhancing the network feature extraction ability, medical clinical experience can also be introduced, and the clinical diagnosis experience of doctors can be integrated into the network structure. None of the above methods fully consider the clinical characteristics of nodules. The use of the clinical characteristics of nodules is the key to improving the performance of the diagnostic network. The aspect ratio of thyroid nodules is important clinical information in the diagnosis of benign and malignant thyroid nodules^22^. In clinical practice, when a nodule has an aspect ratio of greater than 1, doctors will consider the nodule to be malignant. Therefore, based on clinical experience, this paper extract nodular aspect ratio information to enhance the classification performance of the network.

Regarding the issue above, a new network model for benign and malignant thyroid nodule diagnosis is proposed in this paper. The model adopts the diagnostic strategy of localization-classification. The prior design of the localization network is completed by data analysis of ultrasound images so that the localization network can automatically obtain the nodule aspect ratio. At the same time, the attention mechanism is improved and the nodule aspect ratio feature is introduced into the classification module by drawing on clinical experience. Through comparative experimental analysis, it is proven that the proposed network model has better diagnostic performance than the traditional model.

## Material and methods

Regarding the issue above, this paper proposes a new benign and malignant diagnosis network model of thyroid nodules by referring to clinical experience. The model adopts the diagnostic strategy of localization-classification. First, the nodule area image and the aspect ratio information are extracted by locating the nodule of interest. Then nodular region images and uncut ultrasound images are input into a dual-branch network to complete feature extraction. Finally, the image features and aspect ratio features are fused to complete benign and malignant classification.

### Experimental dataset

**Figure 1:**
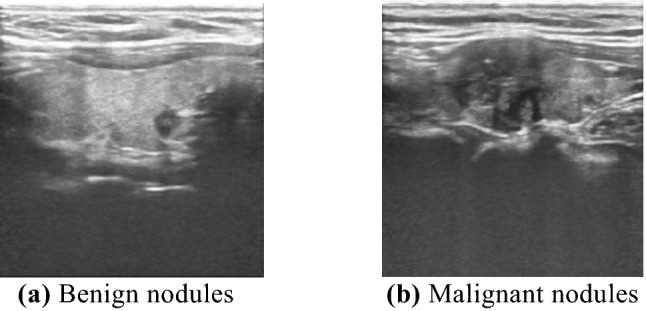
Ultrasonic images of nodules.

The ultrasound data used in the experiment contains 4021 images of thyroid nodules diagnosed in the Inner Mongolia People’s Hospital from October 2017 to December 2020, including 1844 images of benign nodules and 2177 images of malignant nodules. The data were acquired using a GE LOGIQ E9 device, and the images were manually cropped by a physician. All ultrasonic images of nodules were labeled with benign and malignant categories by senior physicians, and the category labels and external rectangular coordinates of nodules were obtained. The data of the ultrasonic images of nodules are shown in Fig. 1. Data have been approved by the local hospital ethics review board, and informed consent from patients was obtained prior to this study. These data were desensitized by doctors and do not contain the patient's private information, only the ultrasound area of the ultrasound image. At the same time, all methods were carried out in accordance with relevant guidelines and regulation. The dataset is randomly divided into training set, test set and verification set according to the ratio of 3:1:1. These sets are completely independent and have no intersection. And the training set and test set are expanded by flipping left and right and up and down. The validation set maintains its data uniqueness.

### New diagnostic model

**Figure 2:**
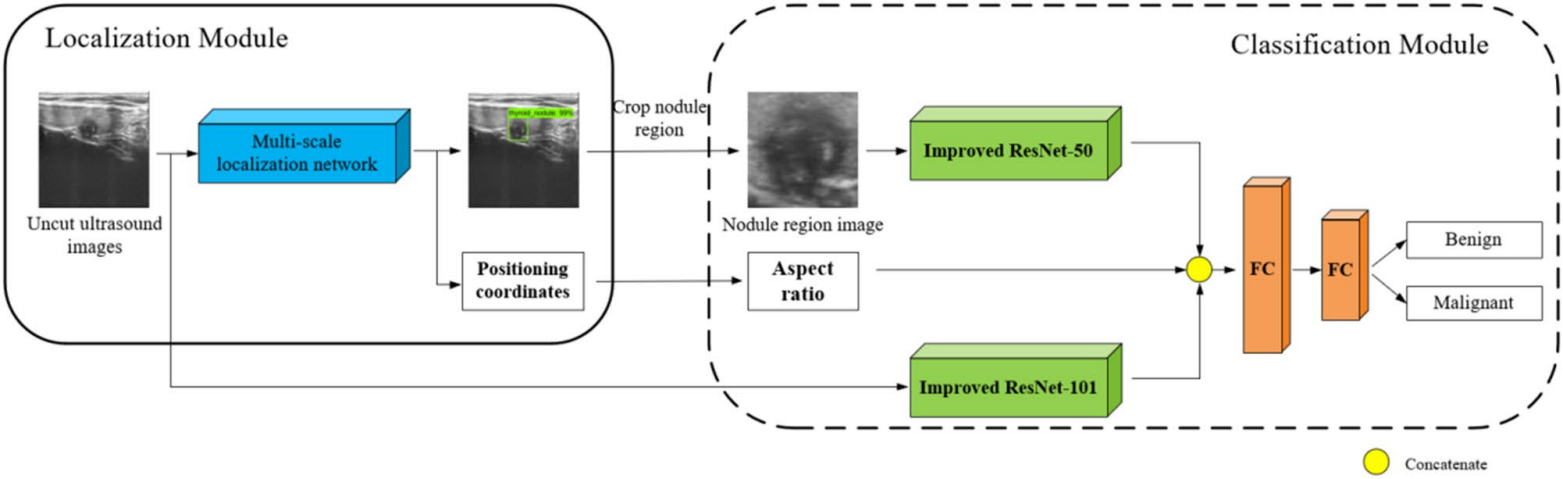
Ultrasound diagnosis network structure for thyroid nodules.

The diagnostic model proposed in this paper is based on a convolutional neural network. The structure of the diagnostic network is illustrated in Fig. 2. The network consists of two main parts: a nodule localization module and a nodule classification module. The nodule localization module adopts a multiscale localization network. The prior design of the parameters of a region proposal network (RPN) was completed by statistical clinical ultrasound data, and the nodule localization effect of the network was enhanced by improving the loss function. Then the nodule area image is obtained by cropping the localization area and extracting nodule aspect ratio information to improve the accuracy of the classification network. The classification network is a dual-branch deep neural network based on global sparse attention and feature fusion. It extracts features from uncut ultrasound images and nodular region images and then fuses the nodule aspect ratio information to complete the classification of benign and malignant nodules. To improve the feature extraction ability of the classification network, this paper proposes and applies the global sparse attention mechanism (GSAM). Compared with the existing attention mechanism, the GSAM has an attention effect in both the spatial and channel dimensions, avoids simple and coarse global pooling, retains more feature information, and effectively improves the feature extraction performance of the network.

#### Localization module

Target detection networks can be divided into two types, namely, one-stage and two-stage, both of which have achieved good experimental results on the Pascal VOC dataset. One-stage series networks are end-to-end detection networks, so the detection speed is faster than that of the two-stage series. However, the two-stage series has higher accuracy due to the generation of finer proposed boxes. Therefore, the representative model of a two-stage series, namely, Faster RCNN^23^, is adopted in this paper as the infrastructure of the localization module. The images contained in the Pascal VOC dataset are natural images, their image features are quite different from those of medical ultrasound images, and the individual size differences among nodules are also large, which makes it difficult for traditional structured detection networks to effectively locate nodules. In research on target detection, multiscale feature pyramid networks (FPN)^24^ are used to detect small target objects. In this paper, the multiscale localization network Faster RCNN + FPN is adopted as the basic framework, and the application of FPN can effectively solve the problem of small nodule localization.

Because of the size differences among thyroid nodules in ultrasonic images, in this experiment, the nodule information of the existing dataset was obtained, and the FPN structure and RPN parameters of the localization module were designed a priori according to this information.

1. Statistical analysis of nodule data

**Figure 3:**
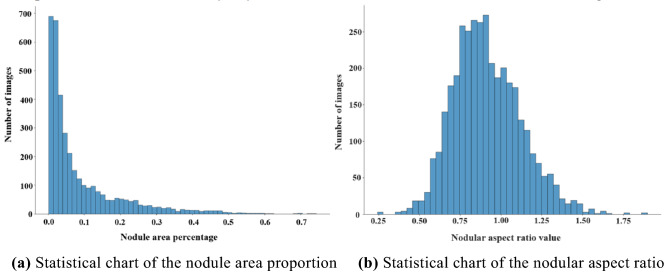
Statistical chart of nodule data.

Ultrasound imaging is related to the location of the acquisition site, so the sizes of nodules in images cannot be directly compared or analyzed. According to the doctor's notes, in this experiment, a statistical analysis on the area proportion of nodules in each image is conducted. The statistical results are shown in Fig. 3a. In this paper, those with a proportion of less than 10% are regarded as small nodules, and those with a proportion of more than 40% are regarded as large nodules. The proportions of the two in the dataset are 74.26% and 2.35%, respectively, and the proportion of the data with an area ratio of less than 5% is 52.65%. The results show that the network should give more attention to the locations of small nodules. This paper uses ResNet^25^ as the feature extraction network of the localization module. The sizes of the output feature graphs of Layer1, Layer2, Layer3, and Layer4 in ResNet are 1/4, 1/8, 1/16, and 1/32 of the input image size, respectively. With the superposition of convolutional layers, the features extracted from the network become more abstract, and the details are gradually lost. Therefore, an FPN structure is needed to enhance the localization of small nodules.

Meanwhile, the numerical distribution of the aspect ratios of nodules in the experimental data is also calculated, as shown in Fig. 3b. According to the statistics, the aspect ratio values of the nodules are mainly distributed between 0.6–1.4, accounting for approximately 94.51% of the samples. However, the aspect ratios of nodules remain unchanged when an image is scaled. Therefore, the aspect ratios of the proposed boxes in the RPN connected by Layer 1 and Layer 2 are set to (3/5,1,7/5), while the aspect ratio span in the RPN connected by Layer 4 is set to (1/4,1,2) to enhance the detection of large nodules.

**Figure 4:**
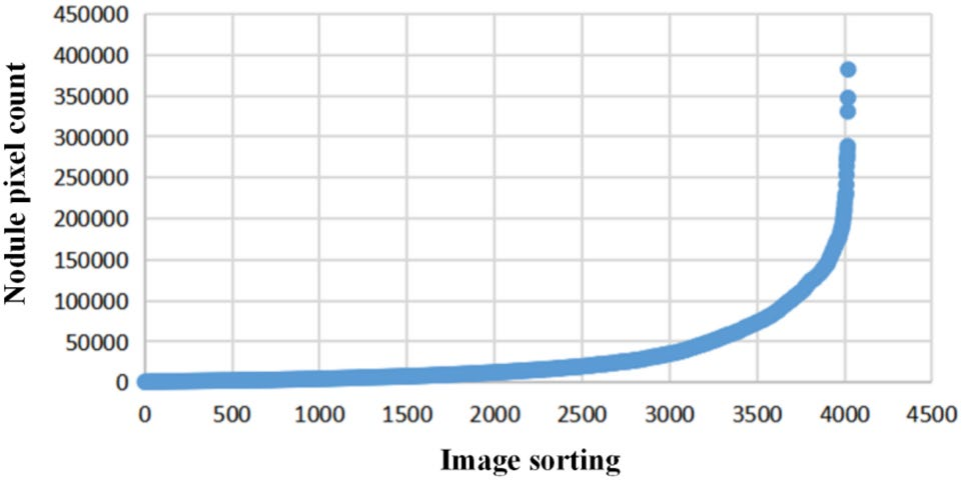
Statistics of the numbers of pixels of nodules.

In this experiment, the number of pixels is used as the standard to calculate statistics on the nodule area size. The images are sorted from small to large according to the number of pixels in the nodule area of each image, as shown in Fig. 4. The small nodules with less than 10,000 pixels in the nodular region account for approximately 45.09%, and the number of large nodules is small. Therefore, an RPN layer targeted at small nodules is constructed using the output features of the first and second residual modules of ResNet50 in this study. The fourth residual block is used to locate large nodules, and the above three residual blocks are used to construct the FPN structure. High-level abstract semantic information is integrated into low-level features to enhance the effect of region generation.

In the experimental data, the minimum number of pixels in the nodular region is 283, and the proportion of small nodules with less than 10,000 pixels is approximately 45.09%. Due to the scale of the input image, the numbers of pixels of such nodular regions mapped in the feature are less than 156. Therefore, proposed boxes of small areas (Sizes) in RPN corresponding to Layer 1 need to be considered, while in RPN 2, the areas of the proposed boxes need to be gradually expanded, and in RPN 3, the size value distribution should be large to detect and locate large nodules. In summary, the anchor point settings of the RPN are presented in Table 1.

**Table 1:** Parameter settings of the RPN anchor points.

Layers	1	2	3
Sizes	(32,64,128)	(32,64,256)	(128,256,512)
Aspect ratios	(3/5, 1, 7/5)	(3/5, 1, 7/5)	(1/4, 1, 2)

(2) Network Structure and Loss Function

**Figure 5:**
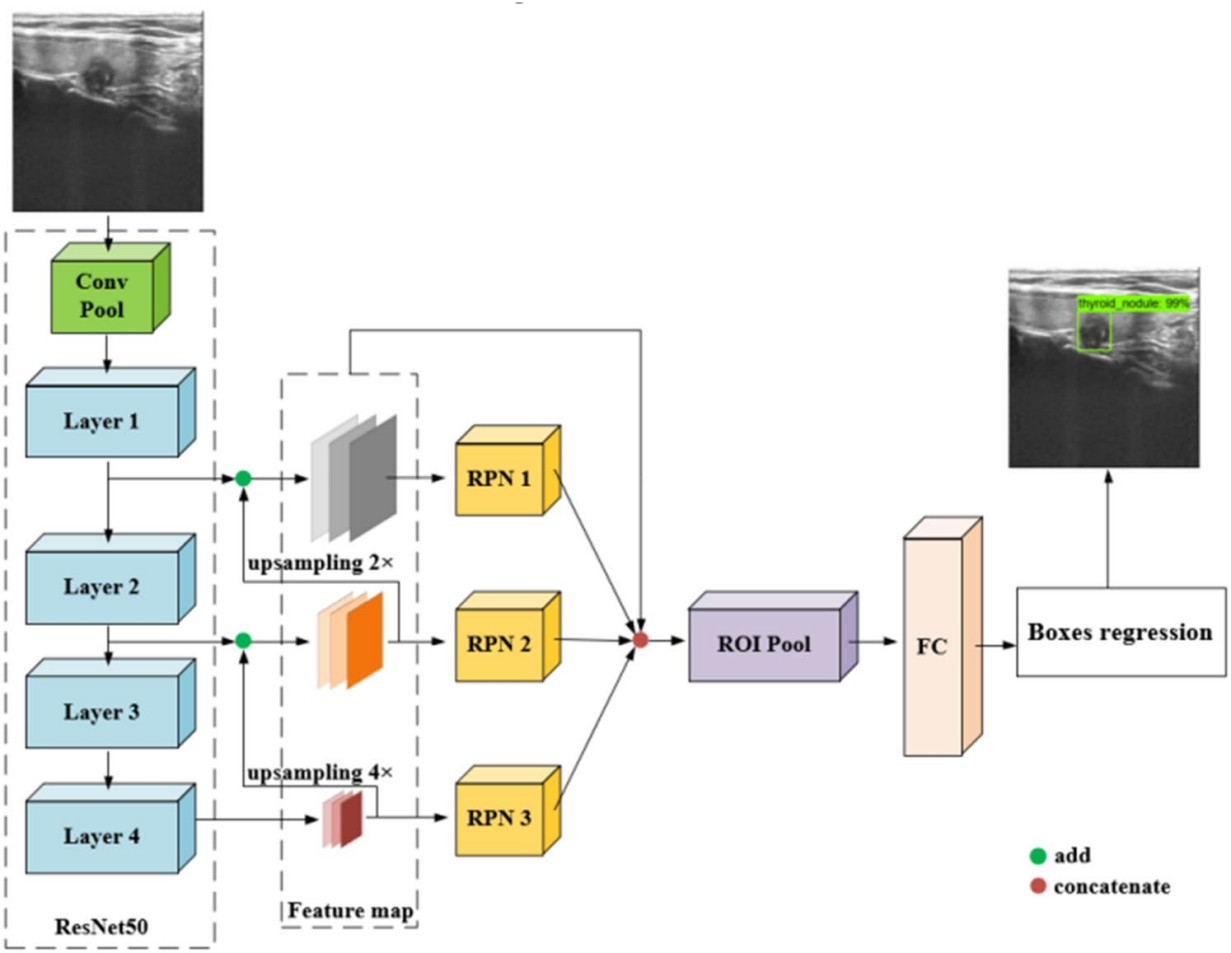
Localization network structure diagram.

Based on the above statistical results, this paper uses Layer1, Layer2 and Layer4 of ResNet50 to form an FPN pyramid for the nodule size distribution to form a multiscale localization network. The specific network structure is illustrated in Fig. 5.

In this paper, the localization network only realizes the function of nodular localization; that is, the network does not classify benign and malignant nodules. Therefore, the classification loss is removed from the RCNN loss function, and the binary classification loss of foreground and background classification is retained and strengthened in the RPN, that is, background classification is conducted before RPN enhancement. The RCNN only completes the positioning task to improve the positioning function of the network.

The loss function of the localization network mainly includes the RPN loss function *L*_*RPN*_ and the Fast RCNN loss function *L*_*RCNN*_. *L*_*RPN*_ calculates the binary classification loss of foreground and background and the candidate box regression loss, as expressed in Formula ( 1):

1 $$L_{RPN} (\{ p_{i} \} ,\{ t_{i} \} ) = \frac{1}{{N_{cls} }}\sum\limits_{i} {L_{{cls{ - }RPN}} (p_{i} ,p_{i}^{*} )} + \lambda \frac{1}{{N_{reg} }}\sum\limits_{i} {p_{i}^{*} L_{{reg{ - }RPN}} (t_{i} ,t_{i}^{*} )}$$

where* L*_*RPN*_ refers to the loss function of the RPN, *L*_*cls-RPN*_ refers to the binary classification loss function of the RPN, *L*_*reg-RPN*_ refers to the candidate box coordinate regression loss function of the RPN, {*p*_*i*_} represents the predicted probability value of the RPN for whether the candidate box contains the target, {*t*_*i*_} represents the candidate box coordinate parameter generated by the RPN, $p_{i}^{*}$ represents the truth label (1 if the target is included and 0 otherwise), $t_{i}^{*}$ represents the coordinate parameters of the real box. λ represents the weight of the regression loss in the loss function, which is used to balance the classification loss and the candidate box regression loss. *N*_*cls*_ denotes the number of candidate boxes, which is set to 256 in the experiment. since the input image size is adjusted to 600 × 600, the size of the obtained feature map is 38 × 38, so λ is set to 6 in the experiment. The binary classification loss *L*_*cls-RPN*_ is expressed in Formula (2):

2 $$L_{{cls{ - }RPN}} (p_{i} ,p_{i}^{*} ) = - \log [p_{i} p_{i}^{*} + (1 - p_{i}^{*} )(1 - p_{i} )]$$

It is easy to know by Eqs. (1) and (2) that when there is no nodule in the ultrasound image, *p*_i_^*^ and *p*_*i*_ are 0 and *L*_*RPN*_ is 0. *L*_*RCNN*_ only calculates the bounding box regression loss, which is used for further regression optimization of the candidate box parameters generated by the RPN, as expressed in Eq. (3):

3 $$L_{RCNN} (\{ t_{i} \} ) = \frac{1}{{N_{reg} }}\sum\limits_{i} {p_{i}^{*} L_{{reg{ - }RCNN}} (t_{i} ,t_{i}^{*} )}$$

#### Classification module

In the diagnosis of benign and malignant thyroid nodules by ultrasound imaging, physicians often give attention to many clinical features of nodules, among which the aspect ratio of nodules is one of the important criteria for distinguishing benign from malignant nodules. In 2021, the team of Fukushima M^26^ cooperated with many medical institutions around the world to study the application value of the aspect ratio of thyroid nodules in the clinical diagnosis of nodules. The experimental results showed that whether the aspect ratio of thyroid nodules is greater than 1 has high application value in the diagnosis of benign and malignant nodules, and the influence of the position and tilt angle of the ultrasound probe on the diagnostic basis can be ignored. Therefore, this paper draws on relevant clinical experience and actively introduces the nodule aspect ratio to improve the network classification performance. Based on the precise positioning effect of the aforementioned positioning network, this paper extracts the aspect ratio information of nodules, fuses it with the features extracted by the convolutional neural network, and inputs the fused features into the fully connected layer to complete the classification and prediction of benign and malignant nodules.

**Figure 6:**
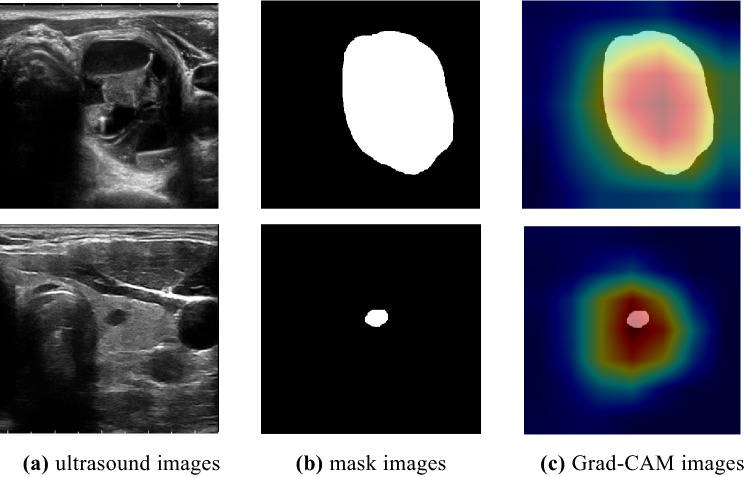
Grad-CAM mapping.

To better explore the nodule ultrasound image features extracted by the classification model, this paper uses Gradient-weighted Class Activation Mapping (Grad-CAM)^27^ in the baseline network experiment of classification to evaluate the model's performance on ultrasound images. Focusing on the region of interest, the results are shown in Fig. 6. The following figure shows the gradient-weighted class activation maps of the two groups of nodule ultrasound classification. Figure 6a presents original ultrasound image, and Fig. 6b presents the nodule mask maps of the ultrasound images obtained according to the physician's annotations. The membrane map is a binary map, and the white area is the nodule area. Figure 6c presents the gradient-weighted class activation maps of the baseline classification network ResNet101 when classifying the images. Figure 6c shows that the classification network mainly focuses on the nodular region in each image, that is, the network mainly extracts the features of the nodular region.

Figure 6 shows that the classification network mainly focuses on the nodular area, but for small-sized nodules, the network also gives attention to the information around the nodules. The small nodule classification performance of the model still needs to be guaranteed, so this paper adopts a dual-branch structure to complete the nodule classification task.

In this paper, a ResNet-based dual-branch classification network is used, and the nodular area image is cropped by using the positioning result. Then, the nodular area image and the uncropped ultrasound image are input into the Net1 branch and Net2 branch of the classification network for feature extraction. To determine the baseline network of the two branches, comparative experiments are conducted on different models of the ResNet series. The results are presented in Table 2, where A represents the use of the uncut dataset and B represents the use of the nodular region clipped dataset. According to Table 2, ResNet50 has the highest accuracy and good sensitivity and specificity for nodular region images. ResNet101 shows moderate specificity but the highest accuracy and sensitivity for uncut ultrasound images of nodules. Therefore, in the classification network, the Net1 branch with nodular region image input adopts the ResNet50 infrastructure, and the Net2 branch with uncut ultrasound image input adopts the ResNet101 infrastructure.

**Table 2:** Comparison of baseline classification networks.

Baseline network	ResNet34		ResNet50		ResNet101		ResNet152	
	A (%)	B (%)	A (%)	B (%)	A (%)	B (%)	A (%)	B (%)
accuracy	73.72	79.19	77.79	**79.82**	**79.59**	78.76	79.17	78.48
specificity	69.25	74.85	**77.91**	75.15	70.86	**75.77**	70.86	70.25
sensitivity	79.26	83.46	77.69	**84.96**	**86.72**	81.20	85.96	85.21

Significant values are in bold.

**Figure 7:**
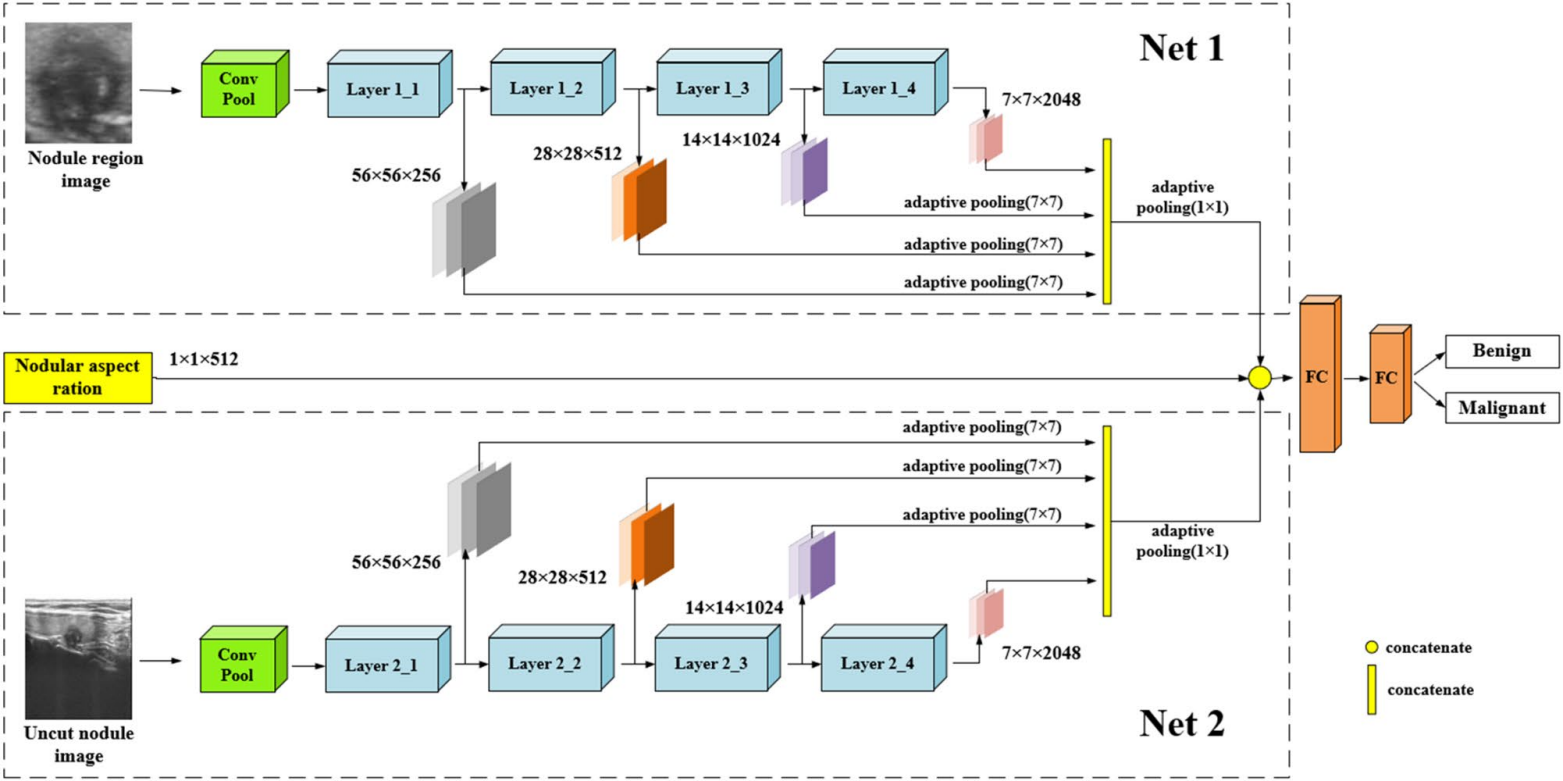
Classification network structure.

To improve the classification performance of the network, three improvements are made in the network structure, and ablation experiments are carried out for each improvement point. (1) The output features of different residual modules are fused. (2) The GSAM is adopted in each residual block of the classification network. (3) The dual-branch and aspect ratio features of nodules are fused to form the final classification feature. The classification module structure is shown in Fig. 7.

#### Global sparse attention mechanism

The attention mechanism enhances the feature extraction ability of the network by increasing the nonlinearity of the feature map in different dimensions. Therefore, this paper improves the feature extraction ability of the network by improving the attention mechanism.

**Figure 8:**
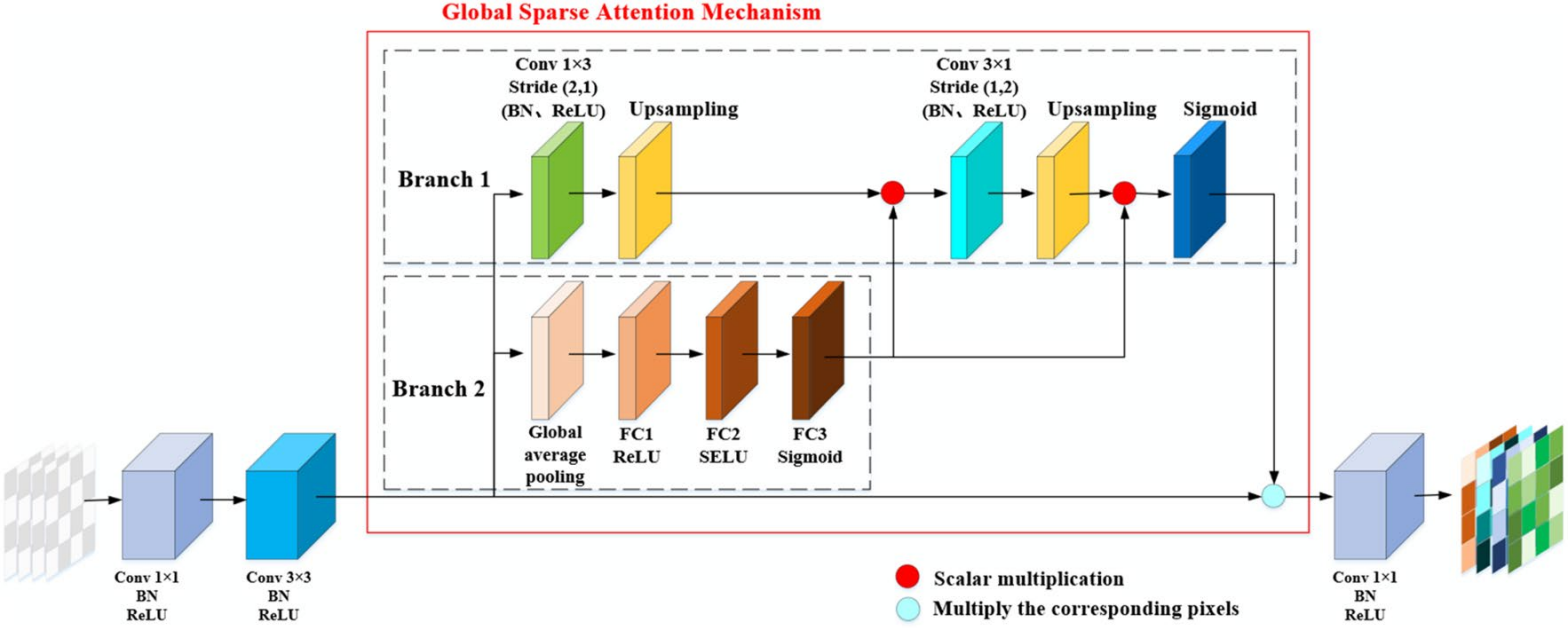
Global sparse attention mechanism.

The network first performs global average pooling or global maximum pooling on the feature map, so that the generated attention only focuses on the global average or maximum value of the feature information, and a large amount of feature information is lost. To effectively use the feature map information to generate attention weights, this paper proposes using the GSAM to improve the attention effect by fusing the spatial dimension and channel dimension information. The improved attention mechanism is illustrated in Fig. 8. It is located in the middle of the 3*3 and 1*1 convolution layers of each block.

In the GSAM, the module gives attention not only to the channel dimension but also to the space dimension. The GSAM has two branches. Branch 2 is the classic channel attention mechanism, which is used for the nonlinear weighting of features in channel dimensions. Branch 1 is a spatial attention mechanism using asymmetric convolution. In branch 1, first, the input features are convolved with the asymmetric convolution kernel Kernel_1 with a size of 1 × 3 and a step size of (2,1), and then the feature size is restored by bilinear interpolation. Then, the scalar weight α generated by channel attention is multiplied by the feature. To enhance the nonlinear expression of features on channel dimensions, the asymmetric convolution kernel kernel_2 with a size of 3 × 1 and step size of (1,2) is used for convolution. The feature dimensions are recovered by bilinear interpolation, and each feature is multiplied by channel attention weight α. Then, the weight values of the spatial dimensions are activated using a sigmoid function. Finally, the corresponding bits are multiplied by the original feature graph.

### Experimental environment

PyTorch is used in the experiment to build a diagnostic network. The training and testing of the localization network and classification network are completed in the following experimental environments: Windows 64-bit OS, Intel Core I9-9900 K CPU, Nvidia Quadro P6000 graphics card, 24G graphics memory, CUDA 10.2, and PyCharm 2020.1.2.

When positioning network training, the Pascal VOC pretraining weight is adopted, a dynamic learning rate is used, the initial learning rate is 0.001, non-maximum suppression (NMS) is set to 0.5, the batch size is set to 12, and the number of epochs is set to 400. The model with the largest mean value on the Dice verification set is saved, and the ratio of the training set, verification set, and test set is 7:2:1. To eliminate accidental factors, the model is trained and tested a total of 5 times. Each time, the dataset is randomly divided according to the ratio of 7:2:1, and the final 5 test results are averaged to obtain the final experimental result of the model.

When training the classification network, the nodular region dataset generated by the uncut dataset and the positioning network is used. First, the ImageNet pretraining weight is used to independently classify Net1 and Net2 without adding aspect ratio information. Then, Net1, Net2, and nodular aspect ratio information are used to build the network, as shown in Fig. 6, for joint training. The data used for training are the dataset of Inner Mongolia People’s Hospital, including 4021 images. The proportion of training set, validation set, and test set is 6:2:2, which is reasonably allocated. The number of epochs is set to 300, and the batch size is set to 32. The binary cross-entropy loss function and a dynamic learning rate are adopted. The initial learning rate is 0.01, and the learning rate is reduced by 90% when the training accuracy does not change for 8 rounds. The model with the highest verification accuracy is saved during training. Similar to the training of the positioning network, to eliminate accidental factors, the model is trained and tested 5 times. Each dataset is randomly divided according to the ratio of 6:2:2, and finally, the 5 test results are averaged to obtain the final experimental result of the model.

### Ethical statement

This study was done in collaboration with the Imaging Department of the Inner Mongolia People’s Hospital. The ultrasound data used in the experiment contains 4021 images of thyroid nodules diagnosed in the Inner Mongolia People’s Hospital from October 2017 to December 2020, including 1844 images of benign nodules and 2177 images of malignant nodules. The data were acquired using a GE LOGIQ E9 device, and the images were manually cropped by a physician. All ultrasonic images of nodules were labeled with benign and malignant categories by senior physicians, and the category labels and external rectangular coordinates of nodules were obtained.

The experimental protocol were approved by ethics committee of Inner Mongolia People’s Hospital, and informed consent from patients was obtained prior to this study. These data were desensitized by doctors and do not contain the patient's private information, only the ultrasound area of the ultrasound image. The ethical research content involved in this research will be managed and engaged in scientific research in strict accordance with relevant national laws, regulations and international practices.

The incidence of thyroid nodules in the population rises year by year, and ultrasound is an important methodology that is currently used in the diagnosis of thyroid cancer because it is noninvasive, safe, and economical. However, ultrasonic images have disadvantages, such as low contrast, low resolution and ease of being polluted by noise, and the rates of missed diagnosis and misdiagnosis by doctors are higher. Deep learning carries out big data training through the construction of a deep convolutional neural network, and the network learns autonomously and is robust. If the project can be implemented in the future, it can realize automatic detection and classification of benign and malignant thyroid nodules, and improve the diagnostic accuracy of doctors.

## Results

### Network evaluation indicators

Since nodule classification is not performed using the localization network in this paper, the localization network is essentially a pixel-level binary network. The higher the coincidence degree between the predicted box and ground-truth box generated by the localization network is, the better the location performance. Therefore, the average Dice evaluation index is adopted in the experiment:

4 $$score = \frac{1}{k}\sum\limits_{1}^{k} {\frac{{2\left| {P \cap \left. T \right|} \right.}}{\left| P \right| + \left| T \right|}}$$

where *P* is the predicted box area, *T* is the ground-truth box area, and *k* is the number of test samples. When the predicted box of the positioning network overlaps with the ground-truth box, the score value is 1, when there is no overlap between the predicted box and the ground-truth box, the score value is 0. The average of all Dice values of the test samples is the network positioning performance evaluation score.

**Figure 9:**
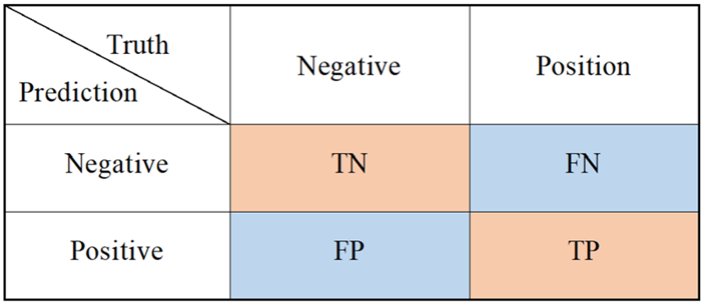
Confusion matrix.

Because benign and malignant data are not strictly 1:1, to better measure the performance of network classification, the accuracy, specificity, and sensitivity generated by the confusion matrix are used as classification evaluation indices. The confusion matrix is a commonly used performance analysis table for binary networks, that describes the classification performance of networks by comparing the predicted categories with real tags. The confusion matrix is shown in Fig. 9. Both the prediction result and the ground-truth labels include 0(negative) and 1(positive). TP indicates that the real label is positive and the prediction result of the model is also positive.TN indicates that the real label is negative and the prediction result of the model is also negative. FP indicates that the real label is negative, but the prediction result of the model is positive. FN indicates that the real label is positive, but the prediction result is negative.

The accuracy, specificity, and sensitivity of the model are calculated as follows:

5 $$accuracy = \frac{TP + TN}{{TP + TN + FP + FN}}$$

6 $$specificity = \frac{TN}{{FP + TN}}$$

7 $$sensitive = \frac{TP}{{TP + FN}}$$

According to the three index formulas, when positive refers to malignant nodules and negative refers to benign nodules, specificity can measure the prediction accuracy of benign nodules, and sensitivity can measure the prediction accuracy of malignant nodules.

### Analysis of the positioning experiment results

**Figure 10:**
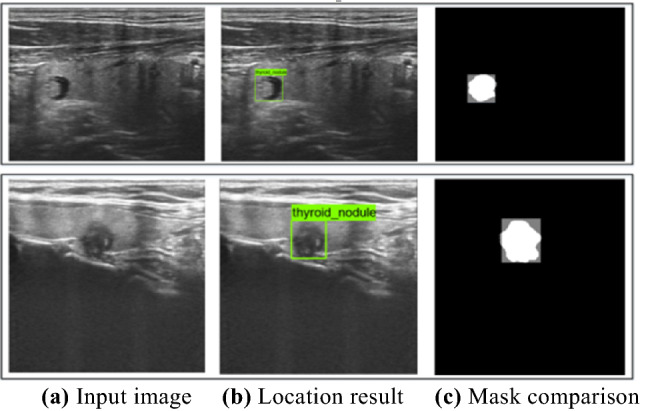
Positioning effect.

The positioning results are shown in Fig. 10. In this paper, the localization network can effectively locate the nodular region in each image, and the detection frame well surrounds the nodule, which can be used to obtain more accurate nodule aspect ratio information.

Figure 10c is a superimposed comparison of the nodule mask and the prediction frame mask. The localization network has good localization performance for nodules. In the experiment, the outer rectangular frames of nodules are first established under the guidance of a senior doctor to generate a real mask, and then the real mask and the mask of the prediction frame are used to calculate the Dice mean value. By calculation, the average Dice index value of the test set is 93.74%. The comparison results of network performance are presented in Table 3, wherein Faster RCNN_1 refers to the traditional Faster RCNN localization network without FPN and Faster RCNN_2 refers to the Faster RCNN network containing FPN.

**Table 3:** Comparison of localization network performance.

Network	Faster RCNN_1 (%)	Faster RCNN_2 (%)	Improved localization network (%)
Average dice	87.43	91.86	**93.74**

Significant values are in bold.

As presented in Table 3, the application of FPN can effectively improve the network positioning performance, and the improvement of the network structure using statistics in this paper further effectively improves the network positioning performance.

### Analysis of the experimental results of classification

To determine whether the cross-level feature fusion, nodular aspect ratio fusion and GSAM proposed in this paper can effectively improve the classification performance of the network, two groups of ablation experiments are conducted. One group is the ResNet50 ablation experiment based on input nodular region images, and the other is the ResNet101 ablation experiment based on input uncut ultrasound images. The experimental results are presented in Table 4 and Table 5.

**Table 4:** Comparison of improvement points based on ResNet50.

Basic model	ResNet50				
Improvement point	Traditional structure (%)	Channel attention (%)	Feature fusion (%)	Nodular aspect ratio (%)	GSAM (%)
Accuracy	79.82	79.58	80.96	**81.24**	81.10
Specificity	75.15	73.93	74.85	**75.98**	75.45
Sensitivity	84.96	84.21	**86.19**	85.71	85.93

Significant values are in bold.

**Table 5:** Comparison of improvement points based on ResNet101.

Basic model	ResNet101				
Improvement point	Traditional structure (%)	Channel attention (%)	Feature fusion (%)	Nodular aspect ratio (%)	GSAM (%)
Accuracy	79.59	81.09	82.07	81.51	**82.34**
Specificity	70.86	75.91	76.35	75.75	**76.65**
Sensitivity	86.72	85.50	86.95	86.45	**87.21**

Significant values are in bold.

Table 4 shows that the traditional channel attention mechanism does not improve the performance of nodular region image classification, presumably because the clipped images contain less information, which makes it difficult for the traditional channel attention of each layer to obtain better global information. The GSAM, on the other hand, preserves a large amount of input feature information and enhances the attention of both the channel and space dimensions to enhance the network's nodular region image classification performance. The use of the nodular aspect ratio and cross-level feature fusion can effectively improve the network classification performance, and the nodular aspect ratio fusion realizes the most obvious improvement of the branch Net1 performance.

As presented in Table 5, when uncut ultrasound data are input, channel attention, cross-level feature fusion, nodular aspect ratio fusion, and the GSAM all improve the ResNet101 classification performance due to increases in image information, among which the GSAM has the most obvious improvement effect on the ResNet101 classification performance.

**Figure 11:**
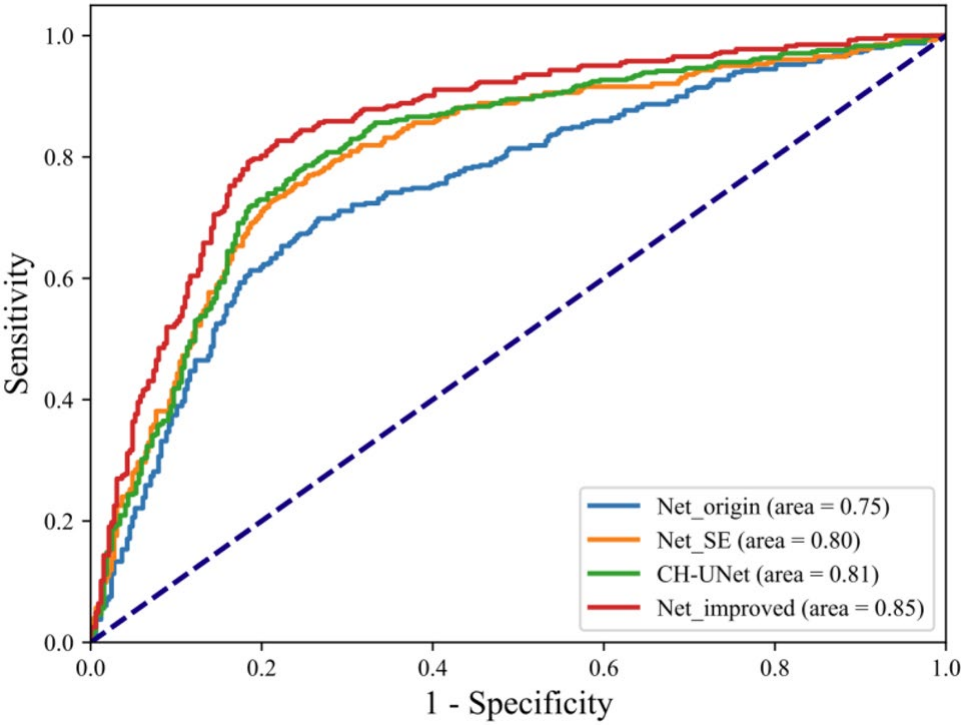
Comparison of ROC curves of four models.

In Table 6, Net_origin refers to the classification performance of a dual-branch classification network without the above three improvements (attention mechanism, cross-hierarchical feature fusion, nodular aspect ratio fusion). Net_SE refers to the traditional attention mechanism based on the fusion of the cross-level feature and nodular aspect ratio. Net_improved refers to the two-channel classification network with the GSAM, cross-level feature fusion, and nodular aspect ratio fusion proposed in this paper. CH-UNet^28^ refers to Classification Head-UNet, the champion model of the Thyroid Nodule Classification Competition TN-SCUI 2020, which was evaluated on the dataset of this paper. As presented in Table 6, the accuracy, specificity, and sensitivity of the classification network proposed in this paper reach 86.34%, 81.29%, and 90.48%, respectively, which are 5.10 percentage points, 4.91 percentage points, and 5.27 percentage points higher than those of the dual-path classification network without improvement and 1.65 percentage points, 0.62 percentage points, and 2.51 percentage points higher than those of Net_SE, which adopts the traditional attention mechanism. Compared with CH-UNet, the specificity is the same, but the accuracy and sensitivity are 1.52 percentage points and 2.77 percentage points higher, respectively, which indicates better benign and malignant nodule classification performance. Also from the comparison of the ROC surfaces of the four networks in Fig. 11, it can be seen that the AUC area of the proposed classification network in this paper is also the largest compared with other networks, reaching 0.85.

**Table 6:** Classification network test comparison.

Classification network	Net_origin (%)	Net_SE (%)	Net_improved (%)	CH-UNet (%)
Accuracy	81.24	84.69	**86.34**	84.82
Specificity	76.38	80.67	**81.29**	**81.29**
Sensitivity	85.21	87.97	**90.48**	87.71

Significant values are in bold.

## Discussion

To solve the problem of benign and malignant classification of thyroid nodules by ultrasound, this paper constructed a diagnosis network using the strategy of localization-classification, which mainly includes a localization module and a classification module.

There are a total of 4021 ultrasound images in the experimental data, which have the characteristics of small samples. To improve the classification performance of the network, an image of the nodule area is obtained by locating the nodule, which reduces the influence of the small sample of experimental data. At the same time, in clinical practice, the aspect ratio characteristics of nodules are also an important basis for diagnosis by doctors, and the aspect ratio characteristics can also be obtained from the nodule localization results. Therefore, the location module is of great significance in this network. In the design of the positioning module, the nodule area distribution and the nodule aspect ratio distribution in the experimental dataset are counted by simulated sampling to design the area generation network parameters of the localization module, and complete the a priori design of the localization network. According to comparative experiments, the prior design based on data statistics can effectively guarantee the localization performance.

The classification network adopts a dual-branch network structure based on global sparse attention and feature fusion. By introducing the GSAM into the ResNet feature extraction module, the channel dimension attention and the spatial dimension attention of the module are enhanced at the same time, which improves the feature extraction ability of the network. Then, the fusion features extracted from the dual-branch network are fused with the aspect ratios of nodules to obtain the final classification features and complete the classification prediction of benign and malignant nodules.

## Conclusions

In this paper, a novel ultrasonic classification method for benign and malignant thyroid nodules was proposed. The parameters of the localization network are a priori designed through experimental data statistics to ensure the accuracy of localization. In the classification network, the nodular aspect ratio information is introduced by referring to the clinical experience of doctors, and the GSAM and high-level-low-level feature fusion are adopted to further improve the classification accuracy. Experimental results show that the proposed method can improve the accuracy of nodule classification more effectively than the traditional method. In future work, we plan to optimize the network structure, try to use a more efficient network structure, and consider using different types of input images such as CEUS. In addition, determining how to extract and apply the clinical features of different types of nodules is also a future research direction.

## Data Availability

The datasets generated and/or analysed during the current study are not publicly available due hospitals are not allowed to disclose but are available from the corresponding author on reasonable request.
